# The Strategy to Use Sugammadex to Reduce Postoperative Pulmonary Complications after da Vinci Surgery: A Retrospective Study

**DOI:** 10.3390/jpm12010052

**Published:** 2022-01-05

**Authors:** Kuang-I. Cheng, Jockey Tse, Tzu-Ying Li

**Affiliations:** 1 Department of Anesthesiology, Kaohsiung Medical University Hospital, Kaohsiung 807, Taiwan; kuaich@gmail.com (K.-I.C.); jockeytse@gmail.com (J.T.); 2 Graduate Institute of Clinical Medicine, College of Medicine, Kaohsiung Medical University, Kaohsiung 807, Taiwan

**Keywords:** postoperative, pulmonary complications, da Vinci surgery

## Abstract

In 2000, the da Vinci Surgery System was approved by the United States Food and Drug Administration for general laparoscopic surgery and it became the first commercially available robotic surgery system. The aim of this study was to identify the incidence of postoperative pulmonary complications (PPCs) in patients undergoing da Vinci surgery and to observe whether the incidence of PPCs was affected by the usage of Sugammadex. Sugammadex is a gamma-cyclodextrin that encapsulates and subsequently inactivates steroidal neuromuscular blocking agents. A retrospective study was conducted on patients who had undergone da Vinci surgery in a single medical center in southern Taiwan during the period from January 2018 to December 2018. We extracted data on patient characteristics, usage of Sugammadex and PPCs for analysis. Three hundred and thirty-three patients were enrolled in the final analysis. While the overall incidence of PPCs was 30.3% (101/333 patients), the incidence of PCC in patients who received Sugammadex (24.2%) was significantly lower than those without (37.3%) (*p* = 0.001). Risk factors that appeared to be closely associated with PCC included age, malignancy, hypertension, chronic kidney disease, blood loss amount and anemia. The use of Sugammadex decreased the risk of PPC. In order to enhance early recovery after da Vinci surgery, the use of Sugammadex to rapidly reverse muscle relaxants may be an appropriate choice.

## 1. Introduction

Robotic-assisted surgery with the da Vinci Surgical System has been an advanced option in neurological, urological, gynecological, cardiothoracic, and numerous general surgical procedures since 2000. The benefits of the Surgical System include less abdominal access trauma, less postoperative pain, a shorter hospitalization period, quicker return to normal functioning and improved cosmetic effects [1,2,3].

The da Vinci Surgical System allows the surgeon’s hand movements to be scaled, filtered, and translated into the precise movements of the EndoWrist instruments (Intuitive Surgical, California, United States) working inside the patient’s body. Any unexpected accidental patient movement will disrupt the whole surgical procedure. Complete or profound paralysis of the patient is required to obtain optimal surgical conditions. In order to profoundly paralyze the patient, most anesthesiologists administer a continuous infusion of muscle relaxant intraoperatively, even though this increases the risk of residual paralysis postoperatively. Patients with a residual neuromuscular blockade may have a higher incidence of adverse respiratory events such as hypoxia and re-intubation in the post-anesthetic care unit (PACU), thereby delaying their discharge [4,5]. Sugammadex is a gamma-cyclodextrin that encapsulates and subsequently inactivates steroidal neuromuscular blocking agents [6]. The use of Sugammadex has been associated with a clinically and statistically significant lower incidence of major pulmonary complications [7]. Nevertheless, whether this effect is also observed in robotic surgery has not been reported. This study aimed to ascertain the incidence and risk factors of postoperative pulmonary complications (PPCs) after da Vinci surgery and determine whether or not the use of Sugammadex could decrease PPCs.

## 2. Materials and Methods

We conducted a retrospective non-interventional study in patients undergoing da Vinci surgery, in a single medical center located in southern Taiwan. This study was approved by the Institutional Review Board of Kaohsiung Medical University Hospital (KMUHIRB-E (I)-20210039, date of approval 26 February 2021), and the trial was conducted according to standards of good clinical practice and the Helsinki Declaration. Patients undergoing da Vinci surgery were enrolled from January 2018 to December 2018. The inclusion criteria included patients aged between 20 and 70 years with an American Society of Anesthesiologists (ASA) physical status classification of I to IV, who were proposed for da Vinci Surgery. The exclusion criteria included difficult intubation under video-laryngoscopy and incomplete documentation in medical records.

Data extracted from the electronic medical records included age, gender, malignancy, tobacco/cigarette use, body mass index (kg/m^2^), ASA physical status, chronic illness such as diabetes mellitus (DM), hypertension, chronic kidney disease (CKD), coronary artery disease history (CAD), asthma, chronic obstructive pulmonary disease and stroke, laboratory data such as hematocrit and creatinine, length of hospital stay, post-anesthesia care unit stay time, blood transfusion units and PPCs.

According to our center’s protocol, all patients undergoing da Vinci surgery receive anesthesia by well-experienced anesthesiologists, with the following medications: induction with fentanyl 1 mcg/kg, thiamylal 5 mg/kg, rocuronium 1 mg/kg, with maintenance of propofol target control infusion (effect-site concentration (Ce) 3.5 mcg/mL) after neuromuscular paralysis. After intubation, anesthesia is maintained with 1.5% sevoflurane and propofol target control infusion (Ce 1.5 mcg/mL), and a continuous infusion of rocuronium at 0.5–0.8 mg/kg/h in order to maintain deep or profound neuromuscular blockade as guided by the post-tetanic count and train-of-four (TOF) count (NMT MechanoSensor, GE Healthcare, Chicago, IL, USA). We check the TOF of each patient every 15 min during the operation to keep the TOF count at zero in order to prevent sudden patient movement during surgery. The rocuronium infusion is stopped after the da Vinci EndoWrist instruments are removed, and the surgeon is ready for wound closure. When the operation has been completed, the patients who chose to use Sugammadex received a fixed dose of Sugammadex of either 2 or 4 mg/kg based on the TOF counts, while the rest receive acetylcholinesterase inhibitor if the TOF ratio is less than 0.9 before tracheal extubation. Acetylcholinesterase inhibitor dosing is based on TOF, that is, TOF of 0.4–0.9 with neostigmine 2 mg (20–30 mcg/kg). TOF showed second twitch (T2) to TOF less than 0.4 with neostigmine 3 mg (30–40 mcg/kg). None of our patients had TOF less than second twitch (T2) at the end of surgery and all the patients were extubated at TOF > 0.9.

### 2.1. Primary and Secondary Outcomes

The primary outcome was to analyze the incidence of PPCs in patients undergoing da Vinci surgery, with the secondary outcome being to ascertain the associated risk factors of PPCs and to evaluate if the use of Sugammadex could decrease the incidence of PPCs. The definition of PPCs is based on the guidelines for perioperative clinical outcomes published by the European Joint Task Force in 2015 (Table 1) [7].

### 2.2. Statistical Analyses

**Figure 1:**
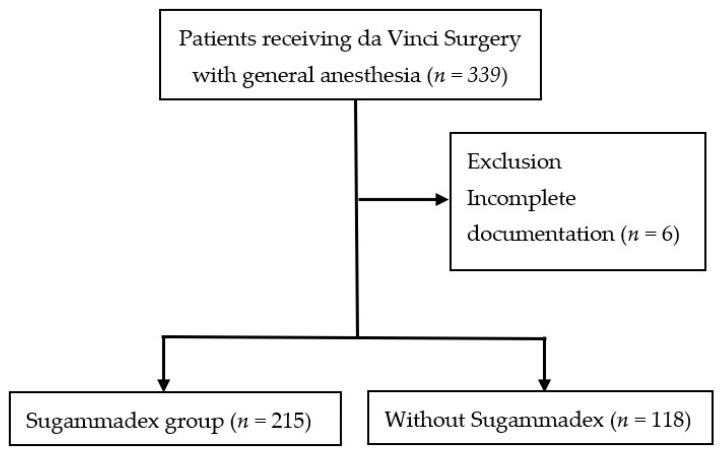
Flow diagram of study group.

In order to determine the associated risk factors of PCC after da Vinci surgery, statistical analysis of continuous variables between groups was carried out by the Student’s *t*-test, with the categorical nominal variables being analyzed by the chi square test. For analyses of whether the use of Sugammadex could decrease PCC rate, patients were categorized into two groups: one group with Sugammadex (Group S, 215 patients); and the other without Sugammadex (Group N, 118 patients). The PPC incidence in the groups were compared with the Student’s *t*-test (Figure 1) The log-binominal model was used to analyze the relationship between PPC and other factors, including patient age, gender, malignancy, cigarette or other tobacco use, body mass index (kg/m^2^), chronic illness (diabetes mellitus, hypertension, chronic kidney disease and stroke history), and laboratory data (hematocrit and creatinine) (Table 2).

## 3. Results

Three hundred and thirty-nine patients were enrolled in the study from January 2018 to December 2018, with six patients being excluded due to incomplete documentation. The majority of the da Vinci surgery in our hospital was radical proctectomy (160/333, 48%, Table 1), followed by radical prostatectomy (39%) and gynecological surgery (13%, hysterectomy or myomectomy). The overall incidence of PPCs was 30.3% (101/333 patients). The most common PPC was respiratory infection (95/333 patients, 28.5%), followed by pneumonia (6/333 patients, 1.8%). No patient suffered from respiratory failure, pneumothorax, bronchospasm or aspiration pneumonitis in this study.

Factors that might increase PPCs include age, lack of Sugammadex usage, malignancy, hypertension, CKD, blood loss amount and anemia. Among all patients, 215 patients received Sugammadex at the end of surgery, while 118 patients did not (Figure 1). The incidence of PPCs in patients who received Sugammadex was significantly lower than those without (24.2 versus 37.3%, *p* = 0.001) (Table 3). Our data showed that the use of Sugammadex could decrease the risk of PPCs and shorten the hospital stay in patients undergoing da Vinci surgery.

Univariate descriptive statistics showed that factors including age, Sugammadex usage, malignancy, hypertension, chronic kidney disease, blood loss amount and preoperative anemia increased PPC incidence after da Vinci surgery (Table 4). To determine the independent predictors of PPC, multivariate risk analyses were performed. After adjusting for age, gender, Sugammadex usage, smoking, malignancy, hypertension, DM, CKD, blood loss, body mass index (BMI) and preoperative hemoglobin level, the only remaining risk factors were malignancy and the use of Sugammadex (Table 5).

## 4. Discussion

Our single-center retrospective observational study showed that the overall incidence of PPCs after da Vinci surgery was 30.3% (101/333 patients). The most common pulmonary complication was respiratory infection (95/333, 28.5%) followed by pneumonia (6/333, 1.8%). The associated risk factors of PPCs include age, malignancy, hypertension, chronic kidney disease, blood loss amount and preoperative anemia. In addition, the use of Sugammadex not only decreased the risk of PPCs but also shortened the hospital stay of patients undergoing da Vinci surgery for malignancy.

The definition of PPCs differs among studies, and its incidence ranges from <1% to 39%. [9,10]. A Multicenter Study by the Perioperative Research Network Investigators found that of 1202 patients who underwent predominantly abdominal, orthopedic, and neurological procedures, at least one PPC occurred in 401 patients (33.4%) [11]. In our study, the PPCs rate was 30.3% after da Vinci surgery, which is similar to this multicenter study, but is relatively high compared to most previous studies [9]. Most of our patients received anesthesia for more than 4 h, with an average anesthesia time of 352.9 ± 115.9 min. Most of them were placed in the Trendelenburg position during surgery. Both the long anesthesia time and surgical positioning might increase the risk of PPCs after surgery.

Sugammadex is a modified γ-cyclodextrin designed as an antagonist of rocuronium and other steroidal neuromuscular blockers [12,13,14]. Currently, numerous studies have shown that Sugammadex provides rapid reversal of rocuronium- or vecuronium-induced neuromuscular blockade with a low incidence of residual or recurrent neuromuscular blockade and is generally well tolerated [15]. This study found that even though all patients were extubated at a TOF ratio > 0.9, patients who received Sugammadex suffered from lesser PPCs. In one multicenter observational matched cohort study of noncardiac surgery, Sugammadex administration was associated with a 30% reduced risk of PPCs, a 47% reduced risk of pneumonia, and a 55% reduced risk of respiratory failure compared to neostigmine [7]. The benefit of Sugammadex on neuromuscular recovery could well extend to the postoperative periods and affect outcomes such as pneumonia and respiratory failure. These advantages were also observed in our study. In our study, we found the incidence of PCC in patients who received Sugammadex (24.2%) was significantly lower than those without (37.3%) (*p* = 0.001).

Malignancy has been validated as a PPC risk factor in previous study [16]. Malignancy, along with other risk factors including ethanol use, current smoker, DM requiring insulin therapy, hypertension requiring medication and a BMI less than 18.5 or ≥ 40 kg/m^2^ increase the incidence of unanticipated early postoperative re-intubation. Unanticipated early postoperative re-intubation in turn is independently associated with a 9-fold increase in mortality [16]. Both poor nutrient status and low albumin level, which are common in cancer patients, are associated with an increased risk of pulmonary complications [10]. As compared with conventional open surgery for viscera malignancy, laparoscopic surgery has the advantages of less tissue damage, lower postoperative pain scores, and lower incidence of PPCs. From our results, the strategy of using Sugammadex in patients undergoing da Vinci surgery could further decrease the incidence of PPCs. As a result, the use of Sugammadex and monitoring of neuromuscular transmission during operations are encouraged.

Anemia, although not statistically significant after adjustment in multivariate analysis, was the only modifiable risk factor before surgery. Preoperative anemia can increase the PCC rate to 1.69-fold that of non-anemic patients (Table 4). The treatment of anemia differs according to the underlying causes, and includes iron supplements or blood transfusion. Clinicians should be cautious of not over-treating anemia, as excess transfusion might cause transfusion-related acute lung injury or transfusion-associated circulatory overload, which will increase pulmonary complications.

Age, hypertension, CKD and blood loss amount have been validated by previous studies as PCC risk factors. These factors were not statistically significant in our study after multivariate analysis and the relatively small sample size may be the reason for such results. Since da Vinci surgery is one of the most expensive self-financed medical procedures in Taiwan, most patients are of working age and are financially sound. Therefore, there were few elderly or young patients in our study, and only 14 out of 333 patients were older than 80 years. Patients from a specific age group with different social backgrounds might affect the results of this study. Further studies with a larger sample size are required.

According to a meta-analysis, restricted fluid infusion can decrease PCC risk in patients with COPD [17]. PPC risk increases by approximately 10% with every 1 mL/kg/h increase in fluid infusion, mainly due to respiratory failure and pleural effusion. Due to the retrospective design, there was no fluid restriction protocol at the beginning of this study. Further prospective studies with proper design are required.

Our study has some limitations. Firstly, the sample size was relatively small and included only 333 patients. Although all anesthesia were conducted by well-experienced anesthesiologists, the risk factors of PCC after da Vinci surgery might be more apparent and clear-cut if a larger sample size was used. Secondly, the study used a retrospective design, so ICD (International Classification of Disease) miscoding or incorrect medical record data might affect the study results. Thirdly, we employed a standard protocol to ventilate patients according to their actual body weight where a tidal volume of 8 mg/kg plus a 4–5 cmH_2_O positive-end expiratory pressure was used. Since different body compositions may require different ventilator settings, we cannot rule out the possibility of inadequate tidal volume and atelectasis caused by our anesthesia protocol. Fourthly, this study included different da Vinci surgeries such as radical proctectomy (48%), radical prostatectomy (39%) and gynecological surgery (13%, hysterectomy or myomectomy). The patient position and surgery time varies according to the surgery type, which might have an influence on the incidence of PCCs; and lastly, a chest x-ray was not arranged for every patient after surgery. According to the “as low as reasonably achievable” principle for radiation exposure, chest x-rays are only arranged for patients with pulmonary symptoms, such as a cough, fever, room air saturation <90% or dyspnea. For patient with obvious clinical symptoms, a chest x-ray with hazy opacity or increased lung infiltration may be categorized as pulmonary infection. We cannot rule out the possibility of pleural effusion combined with respiratory infection. Also, patients who had pulmonary complications without obvious clinical symptoms and signs may be missed during diagnosis. Further well-designed prospective studies are required for a thorough understanding of PCC after da Vinci surgery.

## 5. Conclusions

PPCs in patients undergoing da Vinci surgery are quite common, with possible risk factors including age, malignancy, hypertension, CKD, blood loss amount and preoperative anemia. This study showed that even with adequate recovery of neuromuscular transmission at the completion of surgery, patients who received Sugammadex had a lower risk of respiratory infection and pneumonia. In order to decrease pulmonary complications after da Vinci surgery for malignancy, Sugammadex is a suitable choice for postoperative muscle relaxant reversal.

## Figures and Tables

**Table 1:** Definitions of perioperative clinical outcomes for postoperative pulmonary complications [8].

Complication	Definition
Respiratory infection	Antibiotics for suspected infection with one or more of the following: new or changed sputum, new or changed lung opacities, fever, white blood cell count >12 × 10^9^ liter^−1^
Respiratory failure	Postoperative PaO_2_ < 8 kPa (60 mmHg) on room air, a PaO_2_: FiO_2_ ratio < 40 kPa (300 mmHg), or arterial oxyhemoglobin saturation measured with pulse oximetry <90% and requiring oxygen therapy
Pleural effusion	CXR with blunting of costophrenic angle, loss of sharp silhouette of the ipsilateral hemidiaphragm in upright position, displacement of adjacent anatomical structures, or (in supine position) hazy opacity in one hemithorax with preserved vascular shadows
Atelectasis	Lung opacification with mediastinal shift, hilum or hemidiaphragm shift towards the affected area, with compensatory hyperinflation in adjacent non-atelectatic lung
Pneumothorax	Air in the pleural space with no vascular bed surrounding the visceral pleura
Bronchospasm	Newly detected expiratory wheeze treated with bronchodilators
Aspiration pneumonitis	Acute lung injury after inhalation of regurgitated gastric contents
Pneumonia	CXR with at least one of the following: infiltration, consolidation, cavitation; plus at least one of the following: fever >38 °C with no other cause, white cell count <4 or >12 × 109 liter^−1^, >70 year of age with altered mental status with no other cause, plus at least two of the following: new purulent/changed sputum, increased secretions/suctioning, new/worse cough/dyspnea/tachypnea, rales/bronchial breath sounds, worsening gas exchange

PaO_2:_ Partial pressure of oxygen. FiO_2_: fraction of inspired oxygen. CXR: chest-x-ray.

**Table 2:** Patient characteristics.

Characteristics	Overall (*n* = 333)	Group S^#^ (*n* = 215)	Group N (*n* = 118)	*p* Value
Age, years, Mean (SD)	63.74 (13.02)	61.8 (10.0)	64.8 (13.6)	0.36
Gender, *n* (%)				
Male	197 (59.16%)	120 (55.8%)	77 (65.3%)	0.1
Female	136 (40.84%)	95 (44.2%)	41 (35.7%)	
Anesthesia Time, Minutes, Mean (SD)	352.9 (115.9)	364.8 (105.1)	341.5 (126.3)	0.47
ASA I/II/III/IV	4/208/118/3	3/130/82/0	1/78/36/3	
Body mass index, kg/m^2^, Mean (SD)	24.8 (3.6)	24.8 (3.8)	26.3 (3.2)	0.64
Known Comorbidity				
Diabetes Mellitus, *n* (%)	59 (17.72%)	36 (16.7%)	23 (19.5%)	0.53
Hypertension, *n* (%)	126 (37.84%)	79 (36.7%)	47 (39.8%)	0.58
Chronic Kidney Disease, *n* (%)	10 (3.0%)	4 (1.86%)	6 (5.08%)	0.10
Stroke History, *n* (%)	6 (1.80%)	5 (2.33%)	1 (0.85%)	0.33
Asthma, *n* (%)	5 (1.50%)	3 (1.4%)	2 (1.69%)	0.83
Chronic obstructive pulmonary disease, *n* (%)	4 (1.20%)	3 (1.4%)	1 (0.85%)	0.66
Laboratory Data				
Hematocrit, mg/dL, Mean (SD)	13.3 (1.8)	13.2 (1.72)	13.3 (1.94)	0.58
Creatinine, mg/dL, Mean (SD)	0.96 (0.7)	0.89 (0.47)	1.06 (0.98)	0.20
Surgical Type, *n* (%)				
Radical Prostatectomy	129 (39%)	66 (30.6%)	64 (53.8%)	0.89
Radical Proctectomy	160 (48%)	116 (53.7%)	44 (37.6%)	
Hysterectomy/Myomectomy	44 (13%)	34 (15.7%)	10 (8.5%)	

Group S^#^ (patient using Sugammadex at the end of surgery); Group N (without Sugammadex). *p* < 0.05 means significant difference. ASA: American Society of Anesthesiologists Physical Status Classification. SD: standard deviation.

**Table 3:** Postoperative outcomes.

Postoperative Outcomes	Group S# (*n* = 215)	Group N (*n* = 118)	*p* Value
PACU Stay Time, Minutes, Mean (SD)	77.0 (27.7)	67.6 (15.7)	0.50
Length of Hospital Stay, Days, Mean (SD)	9.2 (4.5)	12.7 (13.5)	0.0007 *
Postoperative Pulmonary Complications, *n* (%)	52 (24.2%)	49 (41.5%)	0.001 *
Respiratory Infection, *n* (%)	51 (23.7%)	44 (37.3%)	0.001 *
Pneumonia, *n* (%)	1 (0.5%)	5 (4.2%)	0.03 *

Group S# (patient using Sugammadex at the end of surgery); Group N (without Sugammadex). * *p* < 0.05 means significant difference. PACU: postanesthesia care unit. SD: standard deviation.

**Table 4:** Risk factors for postoperative pulmonary complications.

Risk Factors	Yes PPC	No PPC	RR (95%CI)	*p* Value
Patient number	101	232		
Age (*n*, %)				0.0125 *
<65	36 (35.64%)	118 (50.86%)	1.00	
≥65	65 (64.36%)	114 (49.14%)	1.5534 (1.0995–2.1946)	
Gender (*n*, %)				0.2355
Female	36 (35.64%)	99 (42.67%)	1.00	
Male	65 (64.36%)	133 (57.33%)	1.2311 (0.8732–1.7355)	
Sugammadex				0.0009 ***
No	49 (48.51%)	69 (29.74%)	1.00	
Yes	52 (51.49%)	163 (70.26%)	0.5824 (0.4233–0.8014)	
Smoking				0.5505
No	97 (96.04%)	219 (94.40%)	1.00	
Yes	4 (3.96%)	13 (5.60%)	0.7665 (0.3202–1.8348)	
Malignancy				0.0010 *
No	8 (7.92%)	62 (26.72%)	1.00	
Yes	93 (92.08%)	170 (73.28%)	3.0941 (1.5796–6.0606)	
Hypertension				0.0296 *
No	54 (53.47%)	153 (65.95%)	1.00	
Yes	47 (46.53%)	79 (34.05%)	1.4299 (1.036–1.9735)	
Diabetes Mellitus				0.9738
No	83 (82.18%)	191 (82.33%)	1.00	
Yes	18 (17.82%)	41 (17.67%)	1.0071 (0.6585–1.5404)	
Chronic Kidney Disease				<0.0001 ***
No	94 (93.07%)	229 (98.71%)	1.00	
Yes	7 (6.93%)	3 (1.29%)	2.4053 (1.5491–3.7348)	
Stroke History				0.8690
No	99 (98.02%)	228 (98.28%)	1.00	
Yes	2 (1.98%)	4 (1.72%)	1.101 (0.3509–3.4546)	
Blood Loss (ml)				0.0083 *
<500	77 (76.24%)	202 (87.07%)	1.00	
≥500	24 (23.76%)	30 (12.93%)	1.6104 (1.1307–2.2935)	
Body Mass Index				0.0504
<25	47 (46.53%)	135 (58.19%)	1.00	
≥25	54 (53.47%)	97 (41.81%)	1.3848 (0.9995–1.9187)	
Preoperative Hemoglobin Level				0.0013 *
Normal	65 (64.36%)	186 (80.17%)	1.00	
Abnormal	36 (35.64%)	46 (19.83%)	1.6953 (1.2286–2.3392)	

Note: Hemoglobin level normal range: male 13–18, female 11–16. * for *p* values < 0.05. *** for *p* values < 0.001. PCC: postoperative pulmonary complications. RR: relative risk. CI: confidence interval.

**Table 5:** Multivariate analysis for risk factors for postoperative pulmonary complications.

Risk Factors	Yes PPC	No PPC	Crude RR (95%CI)	*p* Value	Adjusted ^1^ RR (95%CI)	*p* Value	Adjusted ^2^ RR (95%CI)	*p* Value
Patient number	101	232						
Age (*n*, %)								
<65	36 (35.64%)	118 (50.86%)	1.00		1.00		1.00	
≥65	65 (64.36%)	114 (49.14%)	1.5534 (1.0995–2.1946)	0.0125 *	1.1761 (0.8376–1.6515)	0.3490	1.1591 (0.8767–1.5327)	0.3001
Gender (*n*, %)								
Female	36 (35.64%)	99 (42.67%)	1.00		1.00			
Male	65 (64.36%)	133 (57.33%)	1.2311 (0.8732–1.7355)	0.2355	0.9023 (0.626–1.3006)	0.5816		
Sugammadex								
No	49 (48.51%)	69 (29.74%)	1.00		1.00		1.00	
Yes	52 (51.49%)	163 (70.26%)	0.5824 (0.4233–0.8014)	0.0009 ***	0.6999 (0.5177–0.9464)	0.0204 *	0.7592 (0.586–0.9836)	0.0371 *
Smoking								
No	97 (96.04%)	219 (94.40%)	1.00		1.00			
Yes	4 (3.96%)	13 (5.60%)	0.7665 (0.3202–1.8348)	0.5505	1.0301 (0.4509–2.3534)	0.9439		
Malignancy								
No	8 (7.92%)	62 (26.72%)	1.00		1.00		1.00	
Yes	93 (92.08%)	170 (73.28%)	3.0941 (1.5796–6.0606)	0.0010 *	1.9821 (1.1557–3.3992)	0.0129 *	1.6144 (1.0575–2.4645)	0.0265 *
Hypertension								
No	54 (53.47%)	153 (65.95%)	1.00		1.00		1.00	
Yes	47 (46.53%)	79 (34.05%)	1.4299 (1.036–1.9735)	0.0296 *	1.0463 (0.7478–1.4638)	0.7917	1.0737 (0.8162–1.4125)	0.6111
DM								
No	83 (82.18%)	191 (82.33%)	1.00		1.00			
Yes	18 (17.82%)	41 (17.67%)	1.0071 (0.6585–1.5404)	0.9738	0.8827 (0.5922–1.3156)	0.5399		
CKD								
No	94 (93.07%)	229 (98.71%)	1.00		1.00		1.00	
Yes	7 (6.93%)	3 (1.29%)	2.4053 (1.5491–3.7348)	<0.0001 ***	1.3633 (0.7504–2.4768)	0.3090	1.337 (0.839–2.1307)	0.2219
Stroke History								
No	99 (98.02%)	228 (98.28%)	1.00					
Yes	2 (1.98%)	4 (1.72%)	1.101 (0.3509–3.4546)	0.8690				
Blood Loss								
<500	77 (76.24%)	202 (87.07%)	1.00		1.00		1.00	
≥500	24 (23.76%)	30 (12.93%)	1.6104 (1.1307–2.2935)	0.0083 *	1.2695 (0.8559–1.8828)	0.2355	1.2212 (0.8922–1.6716)	0.2121
BMI								
<25	47 (46.53%)	135 (58.19%)	1.00		1.00			
≥25	54(53.47%)	97 (41.81%)	1.3848 (0.9995–1.9187)	0.0504	1.294 (0.9402–1.781)	0.1137		
Hb								
Normal	65 (64.36%)	186 (80.17%)	1.00		1.00		1.00	
Abnormal	36 (35.64%)	46 (19.83%)	1.6953 (1.2286–2.3392)	0.0013 *	1.3578 (0.9832–1.8751)	0.0633	1.2758 (0.9659–1.6851)	0.0863

Note: Hb (Hemoglobin) normal: male 13–18, female 11–16. CKD: Chronic kidney disease. DM: Diabetes mellitus. BMI: Body mass index. Adjusted ^1^ for age, gender, Sugammadex, Smoking, Malignancy, Hypertension, DM, CKD, Blood loss, BMI, Hb. Adjusted ^2^ for age, Sugammadex, Malignancy, Hypertension, CKD, Blood loss, Hb. * for *p* values < 0.05. *** for *p* values < 0.001. PCC: postoperative pulmonary complications. RR: relative risk. CI: confidence interval.

## Data Availability

The data presented in this study are available on request from the corresponding author. The data are not publicly available due to patient privacy.

## References

[1] Bann S., Khan M., Hernandez J., Munz Y., Moorthy K., Datta V. (2003). Robotics in surgery. J. Am. Coll. Surg..

[2] Smith A., Smith J., Jayne D. (2006). Telerobotics: Surgery for the 21st century. Surg.-Oxf. Int. Ed..

[3] Dobson M.W., Geisler D., Fazio V., Remzi F., Hull T., Vogel J. (2010). Minimally invasive surgical wound infections: Laparoscopic surgery decreases morbidity of surgical site infections and decreases the cost of wound care. Color. Dis..

[4] Murphy G.S., Szokol J.W., Marymont J.H., Greenberg S.B., Avram M.J., Vender J.S. (2008). Residual Neuromuscular Blockade and Critical Respiratory Events in the Postanesthesia Care Unit. Anesthesia Analg..

[5] Butterly A., Bittner E.A., George E., Sandberg W.S., Eikermann M., Schmidt U. (2010). Postoperative residual curarization from intermediate-acting neuromuscular blocking agents delays recovery room discharge. Br. J. Anaesth..

[6] Booij L.H. (2009). Cyclodextrins and the emergence of sugammadex. Anaesthesia.

[7] Kheterpal S., Vaughn M.T., Dubovoy T.Z., Shah N.J., Bash L.D., Colquhoun D.A. (2020). Sugammadex versus Neostigmine for Reversal of Neuromuscular Blockade and Postoperative Pulmonary Complications (STRONGER): A Multicenter Matched Cohort Analysis. Anesthesiology.

[8] Jammer I., Wickboldt N., Sander M., Smith A., Schultz M.J., Pelosi P. (2015). Standards for definitions and use of outcome measures for clinical effectiveness research in perioperative medicine: European Perioperative Clinical Outcome (EPCO) definitions: A statement from the ESA-ESICM joint taskforce on perioperative outcome measures. Eur. J. Anaesthesiol..

[9] Miskovic A., Lumb A.B. (2017). Postoperative pulmonary complications. Br. J. Anaesth..

[10] Busch E., Verazin G., Antkowiak J.G., Takita H., Driscoll D. (1994). Pulmonary Complications in Patients Undergoing Thoracotomy for Lung Carcinoma. Chest.

[11] Fernandez-Bustamante A., Frendl G., Sprung J., Kor D.J., Subramaniam B., Martinez Ruiz R. (2017). Postoperative Pulmonary Complications, Early Mortality, and Hospital Stay Following Noncardiothoracic Surgery: A Multicenter Study by the Perioperative Research Network Investigators. JAMA Surg..

[12] Tarver G.J., Grove S.J.A., Buchanan K., Bom A., Cooke A., Rutherford S.J. (2002). 2-O-Substituted Cyclodextrins as Reversal Agents for the Neuromuscular Blocker Rocuronium Bromide. Bioorg. Med. Chem..

[13] Adam J.M., Bennett D.J., Bom A., Clark J.K., Feilden H., Hutchinson E.J., Palin R., Prosser A., Rees D.C., Rosair G.M. (2002). Cyclodextrin-Derived Host Molecules as Reversal Agents for the Neuromuscular Blocker Rocuronium Bromide: Synthesis and Structure−Activity Relationships. J. Med. Chem..

[14] Bom A., Bradley M., Cameron K., Clark J.K., Van Egmond J., Feilden H., MacLean E.J., Muir A.W., Palin R., Rees D.C. (2002). A Novel Concept of Reversing Neuromuscular Block: Chemical Encapsulation of Rocuronium Bromide by a Cyclodextrin-Based Synthetic Host. Angew. Chem. Int. Ed..

[15] Yang L.P.H., Keam S.J. (2009). Sugammadex. Drugs.

[16] Ramachandran S.K., Nafiu O.O., Ghaferi A., Tremper K.K., Shanks A., Kheterpal S. (2011). Independent Predictors and Outcomes of Unanticipated Early Postoperative Tracheal Intubation after Nonemergent, Noncardiac Surgery. Anesthesiology.

[17] Park S., Oh E.J., Han S., Shin B., Shin S.H., Im Y., Son Y.H., Park H.Y. (2020). Intraoperative Anesthetic Management of Patients with Chronic Obstructive Pulmonary Disease to Decrease the Risk of Postoperative Pulmonary Complications after Abdominal Surgery. J. Clin. Med..

